# Supplementary material for: Brain-Specific Angiogenesis Inhibitor 3 Is Expressed in the Cochlea and Is Necessary for Hearing Function in Mice
Source: Int J Mol Sci. 2023 Dec 4;24(23):17092. doi: 10.3390/ijms242317092 (PMC10707444; doi:10.3390/ijms242317092)
Supplement: Supplementary file 1 [file ijms-24-17092-s001.zip › ijms-2743035-supplementary.pdf]

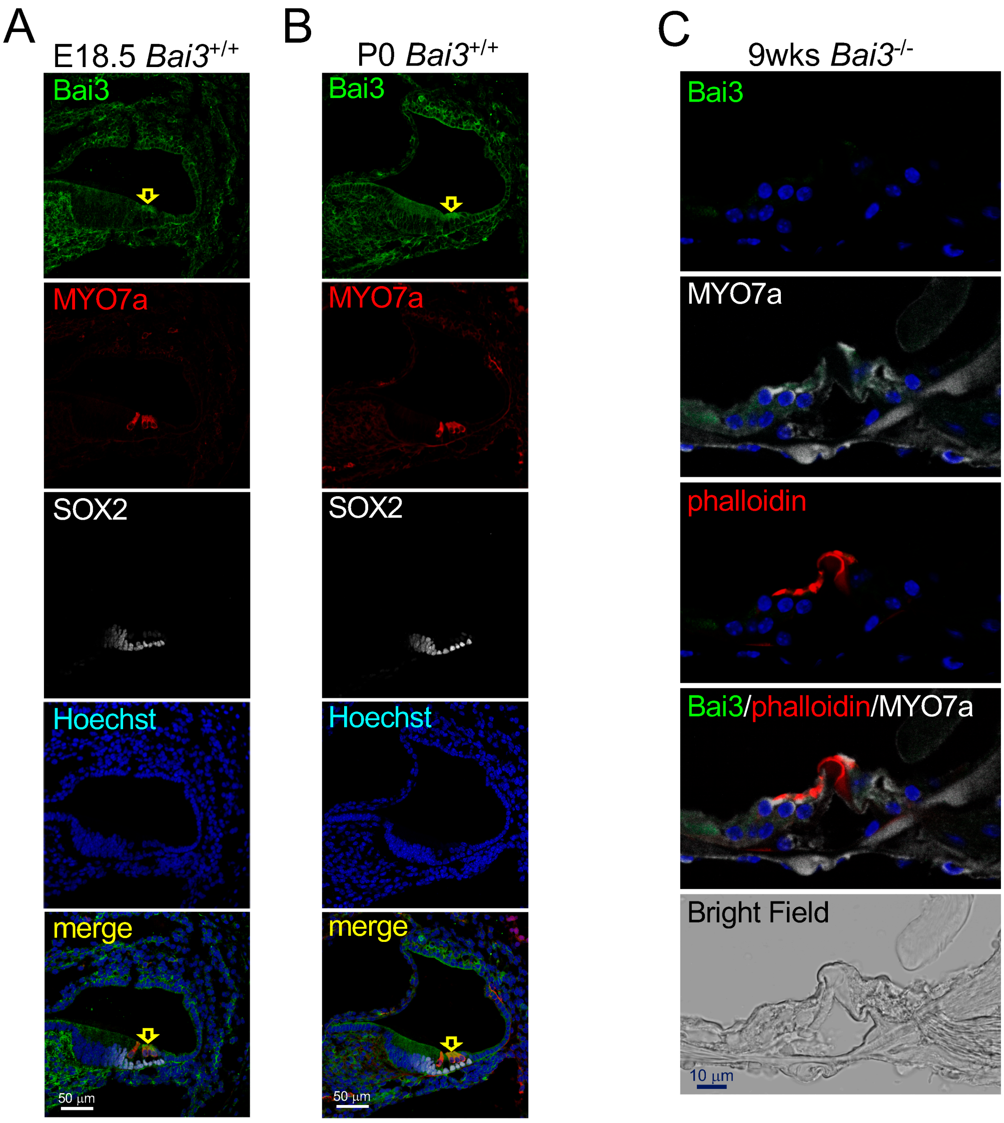

Figure S1: Immunosignals of Bai3 (green) is observed in hair cells of *Bai3*+/+ mice at E18.5 (A) and P0 (B). Tissues are co-immunostained with MYO7a (red), SOX2 (white). Nuclei were stained with Hoechst 33258 (blue). Yellow hollow arrows indicate organ of Corti. In adult *Bai3*+/+ mice, the expression pattern of Bai3 changes from the neonatal stage during which Bai3 is expressed in hair cells, resulting in a pillar cell-specific pattern as shown in Figure 1B. An antibody against the N-terminal extracellular domain of Bai3 was generated in a previous study [7], and its specificity was confirmed by staining the cochlear section of *Bai3*-/- mice. Immunosignals of Bai3 in pillar cells were abolished in adult *Bai3*-/- mice (C). Tissues were co-immunostained with MYO7a (white) and phalloidin (red). Nuclei were stained with Hoechst 33258 (blue).

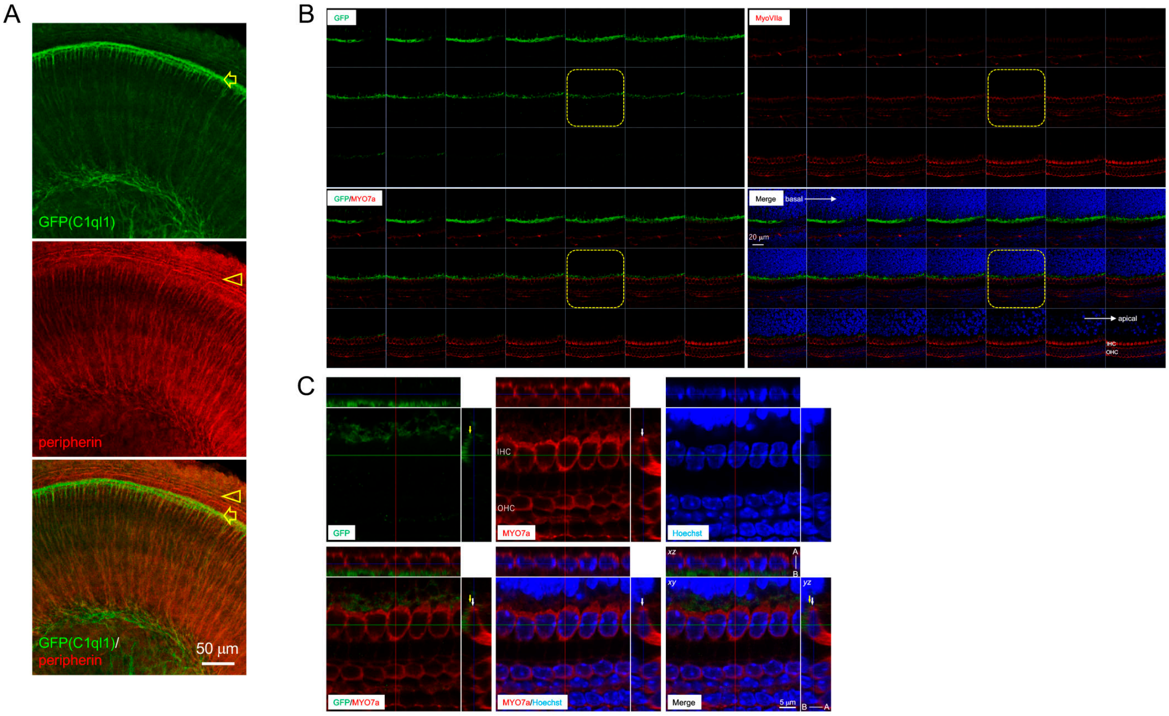

Figure S2: (A) Cochleae of *C1ql1*+/GFP mice at P3 were immunostained using anti-green fluorescent protein (GFP) antibody (green) and anti-peripherin antibody (red). Peripherin-positive neurons innervate the outer hair cells (arrowhead), whereas GFP-positive neurons innervate the inner hair cells (arrows). Each image represents the maximum projection of a confocal z-stack. (B) Images of the sensory epithelium were serially taken along the z-axis from the basal (top left) to apical (bottom right) regions using confocal microscopy. The images marked with yellow dotted lines are enlarged in (C). GFP-positive neurons (green, indicated by yellow arrows) innervate MYO7a-positive inner hair cells (red, indicated by white arrows). A and B in the images denote apical and basal regions of hair cells, respectively. Nuclei were stained with Hoechst 33258 (blue).

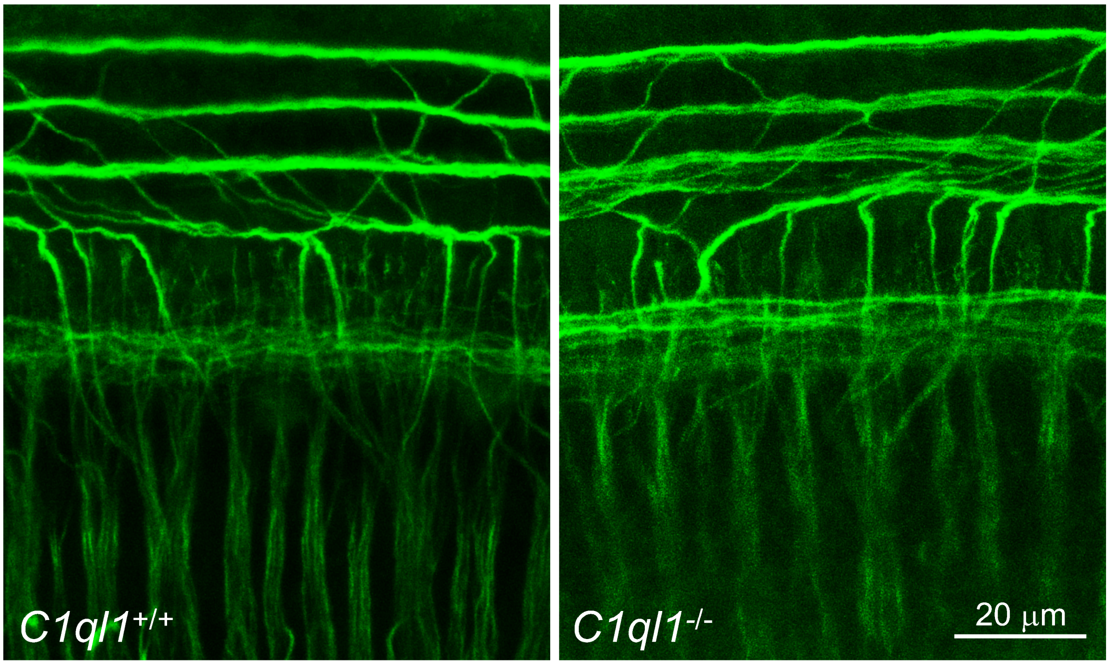

Figure S3: The cochleae of *C1ql1*+/+ and homozygous null mutant *C1ql1*-/- mice at P3 were immunostained using the pan-neuronal marker, NF-H antibody (green). The innervation pattern of NF-H-positive neurons in hair cells was not significantly different between *C1ql1*+/+ and *C1ql1*-/- mice at P3. Each image represents the maximum projection of a confocal z-stack (4.32 µm x 5 sections).

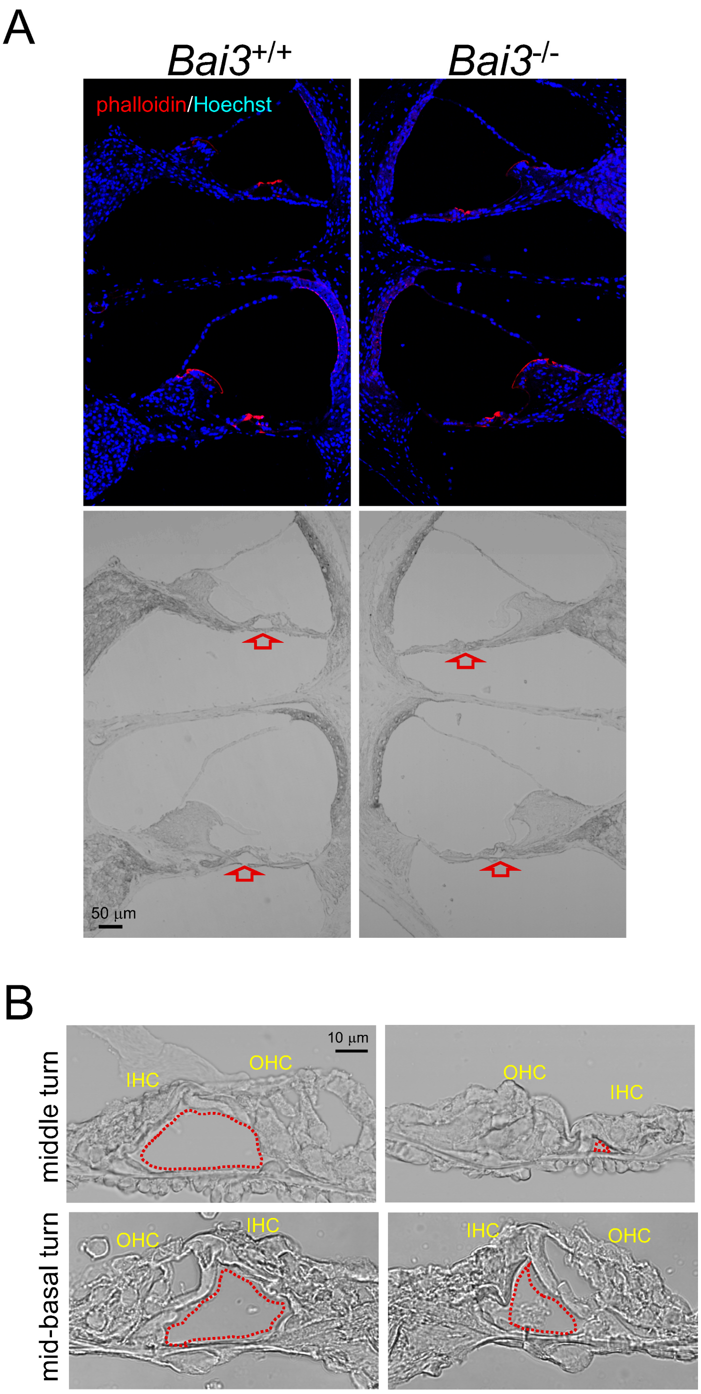

Figure S4: *Bai3*+/+ and *Bai3*-/- cochlea were stained with phalloidin (red in A) and Hoechst (blue in A). Arrows in bright field photo (A, bottom) indicate the location of organ of Corti. (*B*) shows the organ of Corti of *Bai3*+/+ (left) and *Bai3*-/- (right) mice. Histological analysis revealed that tunnel of Corti, marked by dotted red line, are collapsed in *Bai3*-/- mice compared to *Bai3*+/+ mice. Scale bar, 50 µm in (*A*) and 10 µm in (*B*).

Table S1: Experimental conditions for immunostaining.

Supplemental Table 1_Methods for Immunohistochemistry

tissue (mouse age)	antigen retrieval	permeabilization	blocking
E18.5, P0	80℃, 1h	0.3% Triton, 10min	10% NDS/PBST
P3	60℃, 1h	0.3% Triton, 20min	10% NDS/PBS
11 weeks (frozen section)	60℃, 1h	-	10% NDS/PBS
11 weeks (paraffin section)	Microwave 5 min x 3	-	10% NDS/PBST
17 months	-	0.3% Triton, 10min	10% NDS/PBST
